# Two-Component System Response Regulators Involved in Virulence of *Streptococcus pneumoniae* TIGR4 in Infective Endocarditis

**DOI:** 10.1371/journal.pone.0054320

**Published:** 2013-01-16

**Authors:** My Trihn, Xiuchun Ge, Alleson Dobson, Todd Kitten, Cindy L. Munro, Ping Xu

**Affiliations:** 1 VCU Philips Institute of Oral and Craniofacial Molecular Biology, Virginia Commonwealth University, Richmond, Virginia, United States of America; 2 Center for the Study of Biological Complexity, Virginia Commonwealth University, Richmond, Virginia, United States of America; 3 Department of Microbiology and Immunology, Virginia Commonwealth University, Richmond, Virginia, United States of America; 4 The College of Nursing, University of South Florida, Tampa, Florida, United States of America; University of Kansas Medical Center, United States of America

## Abstract

Streptococci resident in the oral cavity have been linked to infective endocarditis (IE). While other viridans streptococci are commonly studied in relation to IE, less research has been focused on *Streptococcus pneumoniae*. We established for the first time an animal model of *S. pneumoniae* IE, and examined the virulence of the TIGR4 strain in this model. We hypothesized that two-component systems (TCS) may mediate *S. pneumoniae* TIGR4 strain virulence in IE and examined TCS response regulator (RR) mutants of TIGR4 *in vivo* with the IE model. Thirteen of the 14 RR protein genes were mutagenized, excluding only the essential gene SP_1227. The requirement of the 13 RRs for *S. pneumoniae* competitiveness in the IE model was assessed *in vivo* through use of quantitative real-time PCR (qPCR) and competitive index assays. Using real-time PCR, several RR mutants were detected at significantly lower levels in infected heart valves compared with a control strain suggesting the respective RRs are candidate virulence factors for IE. The virulence reduction of the ΔciaR mutant was further confirmed by competitive index assay. Our data suggest that CiaR is a virulence factor of *S. pneumoniae* strain TIGR4 for IE.

## Introduction

*Streptococcus pneumoniae* is a gram-positive, alpha-hemolytic bacterial species. It colonizes the upper respiratory tract of humans and is also known to be pathogenic under certain conditions. *S. pneumoniae* causes a number of serious infections in humans, including otitis media, pneumonia, bacteraemia, and meningitis [1]. A large number of strains of *S. pneumoniae* have been identified based on the chemical composition of the polysaccharide capsule, virulence factors, and other antigens [2]. The TIGR4 strain possesses a polysaccharide capsule, which prevents phagocytosis by the host immune system making this strain up to 10^5^ times more virulent than unencapsulated strains such as R6. Virulence may also be attributed to the ability of this bacterium to sense environmental conditions and induce cellular changes that allow strain TIGR4 to thrive and cause invasive diseases. One such disease is infective endocarditis (IE). *S. pneumoniae* belongs to the Mitis group of streptococci, and is classified as a viridans species [3]. While many other viridans streptococci such as *S. sanguinis* and *S. mitis* are more commonly associated with IE, *S. pneumoniae* is also an established IE pathogen, accounting for 1–3% of adult cases [4] and 3–5% of cases in children [5].

IE is an infection of the heart valves or endocardium that affects 10,000–20,000 people every year in the U.S. Lesions of the heart valves or endocardium promote the formation of a sterile vegetation composed chiefly of platelets and fibrin. Certain bacteria, if present in the bloodstream, may colonize the vegetation; bacterial growth enlarges the vegetation, further impeding blood flow and inciting inflammation. Complications from IE can lead to congestive heart failure, aneurysm, or embolic stroke [6]. If left untreated, IE will progress and is uniformly fatal. Treatment is difficult because bacteria embedded in the vegetation are sequestered from antibiotics and most immune defenses. Treatment often involves hospitalization for intensive antibiotic therapy and surgery (including heart valve replacement). Unfortunately, treatment has a high rate of failure, with a mortality rate of approximately 25% and a substantial risk of post-treatment morbidity [6]. Mortality rates of 50% have recently been reported for pneumococcal IE [7]. Unique to pneumococci is a condition called Austrian Syndrome, which consists of meningitis, pneumonia, and endocarditis and is most often observed in middle-aged men with pre-existing conditions such as alcoholism. Although Austrian Syndrome is clinically rare, it is highly aggressive and often fatal because endocarditis is recognized too late [8].

Antibiotic prophylaxis prior to invasive dental procedures has historically been the primary method of IE prevention, because it has been shown that invasive procedures facilitate streptococcal access to the bloodstream [9]. However, more recently, it has become appreciated that most cases of IE occur in the absence of dental manipulation and are therefore not preventable by standard antibiotic prophylaxis provided in conjunction with dental procedures [10]. These findings have led to speculation on links between natural human oral flora and their role in disease. More specifically, studies have been performed to identify virulence factors of streptococci that facilitate infection of the blood and heart. Genes involved in signal transduction through two-component systems (TCSs) have been identified as virulence factors in disease models in various studies [11]–[15], but no published studies have included IE. TCSs are common in bacteria and are composed of a membrane-bound protein called a histidine kinase (HK) and a corresponding cytosolic response regulator (RR) protein. The HK protein senses environmental changes, autophosphorylates a histidine residue, and signals to its corresponding cytosolic RR protein by transferring the phosphate to an aspartate residue. The RR undergoes a conformational change and induces a response by regulating gene transcription. TCSs have been shown to modulate responses such as osmoregulation, chemotaxis, sporulation, photosynthesis, and pathogenicity [16]. In disease, TCSs may regulate expression of virulence factor genes in response to specific environmental cues [17]. We report here for the first time the establishment of an animal model of *S. pneumoniae* IE, and its use to examine the virulence of the TIGR4 strain.

## Materials and Methods

### Animal Ethic

All animals were handled in compliance with all applicable federal guidelines and institutional policies. All of the procedures were approved by Virginia Commonwealth University Institutional Animal Care and Use Committee (IACUC AM10030).

### Bacterial Strain and Medium

*S. pneumoniae* strain TIGR4 was obtained from the American Type Culture Collection (ATCC). All other *S. pneumoniae* mutant strains used were derived from TIGR4 (Table 1). All strains were routinely grown in an atmosphere of 6% O_2_, 80% N_2_, 7% CO_2_, 7% H_2_ generated with an Anoxomat™ system at 37°C in Todd-Hewitt broth (Difco) infused with 0.5% yeast extract, 0.5% glycine, and 11 mM hydrochloric acid (THY+HCl).

**Table 1:** Bacterial strains used in the study.

Strain	Phenotype	TCS^1^	Sources
*S. pneumoniae* TIGR4	Wild type		ATCC BAA-334
Δsp_0083	Erm^r^; Δsp_0083*::ermB*	TCS08	this study
Δsp_0156	Erm^r^; Δsp_0156*::ermB*	TCS07	this study
Δsp_0376	Erm^r^; Δsp_0376*::ermB*	TCS14	this study
Δsp_0387	Erm^r^; Δsp_0387*::ermB*	TCS03	this study
Δsp_0526	Erm^r^; Δsp_0526*::ermB*	TCS13	this study
Δsp_0603	Erm^r^; Δsp_0603*::ermB*	TCS10	this study
Δsp_0661	Erm^r^; Δsp_0661*::ermB*	TCS09	this study
Δsp_0798	Erm^r^; Δsp_0798*::ermB*	TCS05	this study
Δsp_1633	Erm^r^; Δsp_1633*::ermB*	TCS01	this study
Δsp_2000	Erm^r^; Δsp_2000*::ermB*	TCS11	this study
Δsp_2082	Erm^r^; Δsp_2082*::ermB*	TCS04	this study
Δsp_2193	Erm^r^; Δsp_2193*::ermB*	TCS06	this study
Δsp_2235	Erm^r^; Δsp_2235*::ermB*	TCS12	this study
Δsp_1678_Erm	Erm^r^; Δsp_1678*::ermB*		this study
Δsp_1678_Km	Km^r^; Δsp_1678*::aphA-3*		this study

1 TCS cited from Paterson et al. [16].

Erm^r^, erythromycin resistant; Km^r^, kanamycin resistant.

### Construction of Mutants

To study the virulence of TCSs in IE, mutants were made by replacing putative RR genes of TIGR4 with genes (or “cassettes”) encoding resistance to erythromycin (*ermB*) or kanamycin (*aphA-3*). RR genes were identified from the genome annotation. The mutants were constructed by recombinant PCR as previously described [18]. Primers for constructions were designed using Primer3 software (http://frodo.wi.mit.edu/) (Table 2). High-fidelity Taq DNA polymerase (Invitrogen) was used in the PCR reaction mixtures. The PCR conditions to amplify each fragment were 94°C for 1 min., followed by 30 cycles at 94°C for 30 s, 54°C for 30 s, 68°C for 1 min. 30 s, and finally 68°C for 4 min. The approximately 3-kb final linear DNA construct was used for homologous recombination into the TIGR4 genome. The transformation protocol followed for the TIGR4 strain was modified from a previously described protocol for *S. pneumoniae* [19]. Briefly, mid-log phase stock cultures were thawed and diluted 50-fold with media (20 ul culture+980 ul THY+HCl) in conical tubes. Tubes were incubated at 37°C under microaerophilic conditions for 3 hrs to reach O/D_600nm_ = 0.03. To induce competence of cells, 10 N (1 µl) NaOH, 10% (10 µl) BSA, 1 M (1 µl) CaCl_2_, and 2.8 µl CSP-2 (competence stimulating peptide type 2, EMRISRIILDFLFLRKK) was added to each tube and incubated at a 37°C under microaerophilic conditions for 14 min. Mutant donor DNA (50 ng) was then added to tubes and incubated at 37°C under microaerophilic conditions for 90 min. Cells were then diluted 200-fold in media (5 µl cells+995 µl THY+HCl). Transformant bacterial cells were selected by spreading 100 µl of diluted cultures onto antibiotic (10 µg/ml erythromycin or 500 µg/ml kanamycin) selection agar plates. Plates were incubated at 37°C under microaerophilic conditions for 48 hrs. Resultant colonies that were present on selective agar plates and on plates with no antibiotics were counted and the transformation efficiency was calculated.

**Table 2:** Primers used for mutant construction.

Primers	Sequence
SP_0083F1	CATGTGATTCCATACGAACTCTTC
SP_0083R1	CCTTCTCACTATTTAGTCATCCAACTCCCATCTGTCTCTCCTTTGAT
SP_0083F3	AAAGGAGGAAAATCACATGTCCAACTATAAGATAGAGAAACCGAGAGG
SP_0083R3	CATCGGTCACACTGATTGAAAG
SP_0156F1	CCAAAAGCAGGTAGTGGATTTAGTA
SP_0156R1	CCTTCTCACTATTTAGTCATCCAACATACATTTTCTCCCTTTCTACTCA
SP_0156F3	AAAGGAGGAAAATCACATGTCCAACTACCGAAAACAGGTAGAAACTATA
SP_0156R3	GGTCCATTTCATAGAAATTTTTGC
SP_0376F1	GAAAGCTATGACTTGATGCAACACT
SP_0376R1	CCTTCTCACTATTTAGTCATCCAACCCCCATGGCTGACCTACTTATT
SP_0376F3	AAAGGAGGAAAATCACATGTCCAACCGTGGTGTTGGATATACCATGC
SP_0376R3	ATACCATTTGCCTCGTACTATATTTC
SP_0387F1	TCTTTTTATTGTTGGTTTTCAGCAT
SP_0387R1	CCTTCTCACTATTTAGTCATCCAACTTTCATCTTTACTCCTTTATCATTCC
SP_0387F3	AAAGGAGGAAAATCACATGTCCAACCACCATTTGGTGGGGCAAGAGG
SP_0387R3	CCCTCCATTATCACATAAACAGGTA
SP_0526F1	ATGAGAAGACTGGAAGTCTGGTAAA
SP_0526R1	CCTTCTCACTATTTAGTCATCCAACTCTCATCTTCTTACTCTCCCTC
SP_0526F3	AAAGGAGGAAAATCACATGTCCAACGTGTCTGAGGCCATCAATAAAT
SP_0526R3	TATCTGCTCCATATCCTCCTCTTC
SP_0603F1	GCTCTTGAGCAATCTATCTCTGGTA
SP_0603R1	CCTTCTCACTATTTAGTCATCCAACTTTCATACTTTAACTGCTCTCTATTT
SP_0603F3	AAAGGAGGAAAATCACATGTCCAACCGCAATGTTGGTTATAAATTGGAG
SP_0603R3	AAAGTATCTGCAGAACATGGTGATT
SP_0661F1	AGGAAAACTTGAAGAATTTTGTGGT
SP_0661R1	CCTTCTCACTATTTAGTCATCCAACGGTCATGCTCTGCTCCTTTACC
SP_0661F3	AAAGGAGGAAAATCACATGTCCAACCCTCGTCAGTTTAAGAAGGGAG
SP_0661R3	ACAGACAAGAAGAGATGTGACACTG
SP_0798F1	GTTGCTAAATTGGCTCGTCATAACT
SP_0798R1	CCTTCTCACTATTTAGTCATCCAACTATCATGAGAAACTCCTCCTTATT
SP_0798F3	AAAGGAGGAAAATCACATGTCCAACACTTTGCGTAGTGTTGGGTATC
SP_0798R3	ATCATCTCTCCGAGCTAAGTTCA
SP_1227F1	AGTTCCACATCTAGGTGACTGGTAG
SP_1227R1	AAAGGAGGAAAATCACATGTCCAACCGCCGTGGTGTAGGGTATTACA
SP_1227F3	CCTTCTCACTATTTAGTCATCCAACTTTCATATGTTCACCTTTTTCTCTAC
SP_1227R3	AGCCATTTCCAGTATTGCTTTTAAC
SP_1633F1	TAGATAGATAAAGGCCAAGTCCAGA
SP_1633R1	AAAGGAGGAAAATCACATGTCCAACACCAAGAAAGGAATAGGGTACG
SP_1633F3	CCTTCTCACTATTTAGTCATCCAACGTGCATGCGCTTCTCCTTTTCC
SP_1633R3	ATAATGGAACCTTGTGGAATGAATA
SP_2000F1	GAGCGAATTTTATCTGTCAAGTGAT
SP_2000R1	AAAGGAGGAAAATCACATGTCCAACAATATCGCGAAAGAATCTGGTTG
SP_2000F3	CCTTCTCACTATTTAGTCATCCAACTTTCATCTACTTTCTCTCTTATAAA
SP_2000R3	CTGGTTTTTCTTTTTCCTATCCAAT
SP_2082F1	GATATTTCTGCGACTCATTTTGAAC
SP_2082R1	CCTTCTCACTATTTAGTCATCCAACTGTCATCTATTATCTCCTATTGGT
SP_2082F3	AAAGGAGGAAAATCACATGTCCAACGGTTATGGTTATAACTTCAAGGAG
SP_2082R3	AGATGCTCAACAATATGCTCAAGAC
SP_2193F1	CTAATAAGTGGCTCATCTGGTCAAT
SP_2193R1	AAAGGAGGAAAATCACATGTCCAACGTGAAAAATGTTGGGTATAAGATTAG
SP_2193F3	CCTTCTCACTATTTAGTCATCCAACGTTCATCTCTCTCCCTTTCTAC
SP_2193R3	CAAGCTTCAGCTCTGAAATTGTTA
SP_2235F1	AAATTGCTTTCCATTCTTTAAACTT
SP_2235R1	AAAGGAGGAAAATCACATGTCCAACGATATTTTAGAGAAAAAATCTCAAAAGT
SP_2235F3	CCTTCTCACTATTTAGTCATCCAACAACTTTCATTCAAATTCCCTCTTAAA
SP_2235R3	GCCTATTTTGACAAGGACTACCTTT
SP_1678F1	CTGGAATGACACCTTCGTATTTTT
SP_1678R1_Erm	AAGGAGGAAAATCACATGTCCAACTTACAAAGAAAAATGATGGAGGAG
SP_1678F3_Erm	CCTTCTCACTATTTAGTCATCCAACGAACAAATCTATTTTTTCTTTTGGAC
SP_1678R3	GTATCAAAGACGAGGCAGTAGCAT
SP_1678R1_Km	GTTTTAGTACCTGGAGGGAATAATGTTACAAAGAAAAATGATGGAGGAG
SP_1678F3_Km	GCCATTTATTCCTCCTAGTTAGTCAGAACAAATCTATTTTTTCTTTTGGAC
Erm_F	GTTGGATGACTAAATAGTGAGAAGGAGTGATTACATGAACAA
Erm_R	GTTGGACATGTGATTTTCCTCCTTTTTATTTCCTCCCGTTAAATAATAG

### Growth of Mutants

Growth rates of mutants were determined as follows. Each mutant strain from frozen stocks was inoculated into 5 ml THY+HCl and incubated at 37°C under microaerophilic conditions overnight. Overnight cultures were diluted 100-fold into fresh media (56 µl culture+5.54 ml THY+HCl). A total of 200 µl of each mutant culture was pipetted in triplicate into wells of 96-well plates so that each plate had repeats of every mutant. One plate was made for every time point to be measured for the growth curve. Each plate was incubated in separate Anoxomat™ jars at 37°C under microaerophilic conditions. At each time point, a corresponding plate was removed from incubation and the cell growth was measured using the FLUOstar plate reader (BMG Technologies) at OD_600nm_. After 10 hrs of measurements, a growth curve using the average of the hourly repeats for each mutant was constructed from the data collected.

### Virulence Assessment in the Rabbit Endocarditis Model

IE virulence was investigated using a standard rabbit model, employed previously for investigation of IE virulence in oral streptococci [20], [21]. Briefly, male specific-pathogen free, New Zealand White rabbits weighing 3 to 4 kg were used. To induce valve damage, the right internal carotid artery of each anaesthetized animal was nicked, and a catheter was inserted through the opening and threaded until resistance was met, indicating contact with the aortic valve or left ventricle. The catheters was then tied off and sutured in place, and the incision closed. *S. pneumoniae* TIGR4 from a single colony on BBL trypticase soy agar with 5% sheep blood (TSA II) was inoculated into fresh brain heart infusion (BHI) medium and cultured microaerophilically at 37°C overnight. The overnight culture was diluted 10-fold with pre-warmed fresh BHI and cultured at 37°C for 3 h. After centrifugation and washing with PBS, bacterial cells were adjusted to OD_660_ of 0.8 and 0.5 ml of bacterial cells was inoculated into the peripheral ear veins of rabbits catheterized two days prior. The day after inoculation, rabbits were sacrificed and heart valve vegetations were collected, homogenized, serially diluted, and spread onto TSA II agar plates for bacterial enumeration. For competitive index (CI) assays of pooled mutants, the same inoculum prepared for in vitro studies (OD_660nm_ = 0.8 or 2×10^8^ CFU/ml of 14 mutants) was loaded at 0.5ml volume into syringes for injection into the ear vein of four rabbits. Other procedures were as described above. Finally, the harvested heart valve vegetations from sacrificed rabbits were homogenized and used for qPCR analysis.

Additional competition experiments were performed as previously described [20], [21]. Bacterial cells of a single Erm^r^ mutant and a Km^r^ control strain were mixed in equal numbers for inoculation. This inoculum was injected into the ear vein of three catheterized rabbits to evaluate the relative fitness in IE. Bacteria were recovered from infected heart valves 20 hrs later as above, diluted, and enumerated on selection plates with either Km or Erm [20]. The colony counts were applied to the CI formula (mutant/control ratio of the recovered cells divided by the mutant/control ratio of the inoculum) and significance was calculated by one-sample t test.

### Real-time PCR for Quantitation of Pneumococci in Rabbit Vegetations

Primers of approximately 25 bases for qPCR were designed using Primer3 software. The forward primer was complimentary to the *ermB* sequence (or universal primer), while the reverse primer was complimentary to the specific sequence of the downstream fragment of each mutant (or gene-specific primer). Bacterial genomic DNA from the pooled inoculum and the infected rabbit vegetations was isolated using a DNeasy Blood & Tissue Kit (Qiagen) and used as the template for qPCR for each of the 14 mutants. The appropriate amount of genomic DNA was obtained by 10-fold serial dilutions for PCR amplified products. We finally used 2 µl of genomic DNA as template, 1 µl dH_2_O, 1 µl 10 mM common (forward) primer, 1 µl 10 mM specific (reverse) primer and 5 µl 2× PCR Master Mix (Applied Biosystems) in our qPCR reaction mixture. The threshold cycle (C_T_) of each mutant and the control before and after injection into animals were determined in qPCR assay as described previously [22]. We defined qPCR-CI using C_T_ for virulence determination instead of the regular CI using bacterial CFU. The equation of qPCR-CI was as follows.
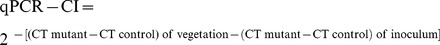



### DNA Sequence Analysis and Database Search

The PCR amplicons were sequenced with an ABI sequencer at the Nucleic Acids Research Facilities of Virginia Commonwealth University. DNA sequences were analyzed with SeqMan II software. Sequences were searched against the completed *S. pneumoniae* genomes with BLAST to confirm the correct TCS gene mutants.

## Results

### Virulence of *S. pneumoniae* in Endocarditis

We set out to examine the virulence of *S. pneumoniae* using a rabbit endocarditis model as previously described for *S. sanguinis* [20], [21]. As before, this model employed catheterization to induce minor damage to the aortic valve, making the animals susceptible to infection. Two days following surgery, about 10^8^ CFU of bacterial cells were inoculated into an ear vein. The following day, animals were euthanized and the infected regions (called “vegetations”) were surgically removed, homogenized, and plated for bacterial enumeration. The log_10_ CFU from vegetations of three rabbits inoculated with *S. pneumonia* TIGR4 were 8.04, 6.69 and 7.65 (mean ± SD = 7.46±0.69), respectively. The infection was similar to that of *S. sanguinis*, which has a typical recovery of 10^7^–10^8^ CFU per animal using similar experimental conditions [20], [21]. This result indicated that *S. pneumoniae* TIGR4 is virulent in our IE animal model. This animal model could therefore be used to study *S. pneumoniae* virulence in IE.

### Construction of TCS Gene Deletion Mutants

To construct TCS gene mutants, we identified all TCS genes in *S. pneumoniae* TIGR4 strain by comparing streptococcal genomes [23]. There were 13 putative HKs and 14 putative RRs, including 1 orphan RR (SP_0376). We also randomly selected a gene, SP_1678, encoding a hypothetical protein that we used as a control in subsequent experimentation.

Recombinant PCR site-directed mutagenesis [18] was used to construct mutants for genes of interest. Each recombinant PCR amplicon contained three fragments–the upstream region of the target gene, an antibiotic resistance cassette, and the downstream region of the target gene. The recombinant PCR amplicons were transformed into competent TIGR4 cells. Following the protocol described by Bricker (1999), TIGR4 transformation efficiency reached 0.02%. After several attempts, we obtained all TCS deletion mutants except for RR SP_1227. No colonies grew for the SP_1227 mutant in multiple transformations. Sequence comparison showed that this gene was an ortholog of an essential gene, SSA_1565, of *Streptococcus sanguinis*. This gene, also known as *vicR*, has been previously reported to be essential for *S. pneumoniae* growth [24]. We therefore concluded that SP_1227 was essential in TIGR4. All mutants were confirmed by colony PCR and DNA sequencing analysis. We previously used an identical approach for the systematic deletion of every non-essential gene in the chromosome of the closely related species *S. sanguinis* [18]. The results of that study indicated that the mutagenesis procedure efficiently and reliably produced non-polar mutants in non-essential genes. In addition, the procedure was used to identify eight essential genes in *S. pneumoniae* TIGR4 [18].

### Examination of Mutant Growth in vitro

**Figure 1:**
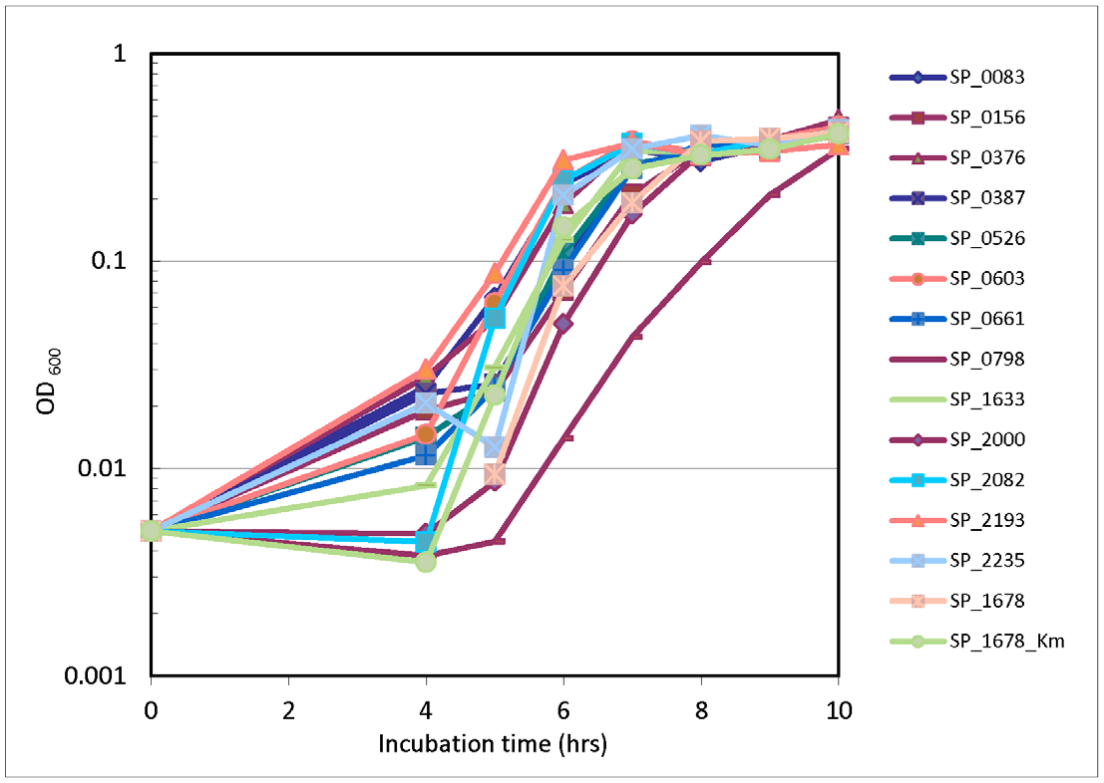
*In vitro* growth of RR mutants. Growth of all 13 RR mutants with Erm-resistance cassettes, and control mutant Δsp_1678 with Erm- and Km-resistance cassettes. Bacterial cultures were individually grown at 37°C in microaerophilic conditions over the course of 10 hrs. Growth was measured by OD readings at 4 hrs and every hour thereafter on a FLUOstar plate reader at 600 nm.

As a preliminary step and reference for further study in our *in vivo* IE model, in vitro experiments to elucidate growth rates of our mutants were conducted. After measuring growth of mutants inoculated individually, we found that all the RR mutants grew at similar rates to the control mutant, Δsp_1678, except for RR SP_0798 (Fig. 1), which grew slightly slower than the other strains from 6–10 hr. It reached a similar OD value after 10 hrs.

### Identification of Virulence-related TCS by qPCR

In accordance with ethical principles to minimize the number of animals needed to generate valid results, we employed a pooling strategy in which 13 candidate mutants were combined together for infection of animals. This approach has been used previously [20] except that mutants were monitored by qPCR rather than DNA hybridization. All mutants were co-inoculated along with our control mutant into four rabbits in the endocarditis model. After necropsy, the pooled bacterial cells were collected from the vegetation of each animal. Bacterial genomic DNA was isolated and subjected to quantitative PCR in triplicates using primers specific for each of the 14 mutant loci. A comparative C_T_ method of qPCR [22] was used to identify virulence genes by comparing amounts of genomic DNA of each mutant with the control. Levels of genomic DNA for each mutant relative to the control in the pool were determined before and after injection into animals. Potential virulence factors were identified based on the qPCR-CI (See Materials and Methods). We analyzed the data gathered from qPCR-CI for each mutant from four rabbits. The analysis (Table 3) indicated that several mutants had qPCR-CI values significantly (P<0.01) less than 1.0, including Δsp_0376, Δsp_0798, Δsp_1633, and Δsp_2082. The mutants Δsp_0376 and Δsp_0798 were significantly lower than the others, suggesting the greater importance of these two genes in IE infection. SP_0376 is an orphan response regulator without a neighboring histidine kinase gene. SP_0798 encodes the *ciaR* gene, which has previously been reported to be involved in systemic virulence in mouse models [11], [16].

**Table 3:** Endocarditis virulence of mutants determined by qPCR-CI.

Mutant	CI (Mean^1^± SD)	P value^2^	Protein name	TCS family	TCS	Virulence^3^
Δsp_0083	2.00±1.37	0.15	DNA-binding response regulator	Pho	TCS08	y
Δsp_0156	0.47±0.26	0.04	DNA-binding response regulator	Lyt	TCS07	y
Δsp_0376	8.57E-05±1.7E-05	7.70E-06	DNA-binding response regulator	Pho	TCS14	y
Δsp_0387	0.66±0.75	0.72	DNA-binding response regulator	Nar	TCS03	n
Δsp_0526	1.01±0.29	0.79	response regulator BlpR	Agr	TCS13	y
Δsp_0603	1.04±0.56	0.68	DNA-binding response regulator VncR	Pho	TCS10	n
Δsp_0661	0.98±0.36	0.83	DNA-binding response regulator	Lyt	TCS09	y
Δsp_0798	0.0026±0.0014	7.40E-10	DNA-binding response regulator CiaR	Pho	TCS05	y
Δsp_1633	0.44±0.16	0.0061	DNA-binding response regulator	Pho	TCS01	y
Δsp_2000	2.36±1.37	0.11	DNA-binding response regulator	Nar	TCS11	n
Δsp_2082	0.33±0.21	0.01	response regulator	Pho	TCS04	y
Δsp_2193	3.75±1.72	0.04	DNA-binding response regulator	Pho	TCS06	y
Δsp_2235	0.95±0.35	0.95	response regulator ComE	Agr	TCS12	y
SP_1227	Essential gene		DNA-binding response regulator	Pho	TCS02	y

1 Mean, geometric mean.

2 P values determined by one-sample t test with false discovery rate control.

3 Virulence cited from Paterson et al. [16].

### Confirmation of Virulence Reduction of the *ciaR* Gene Mutant

**Figure 2:**
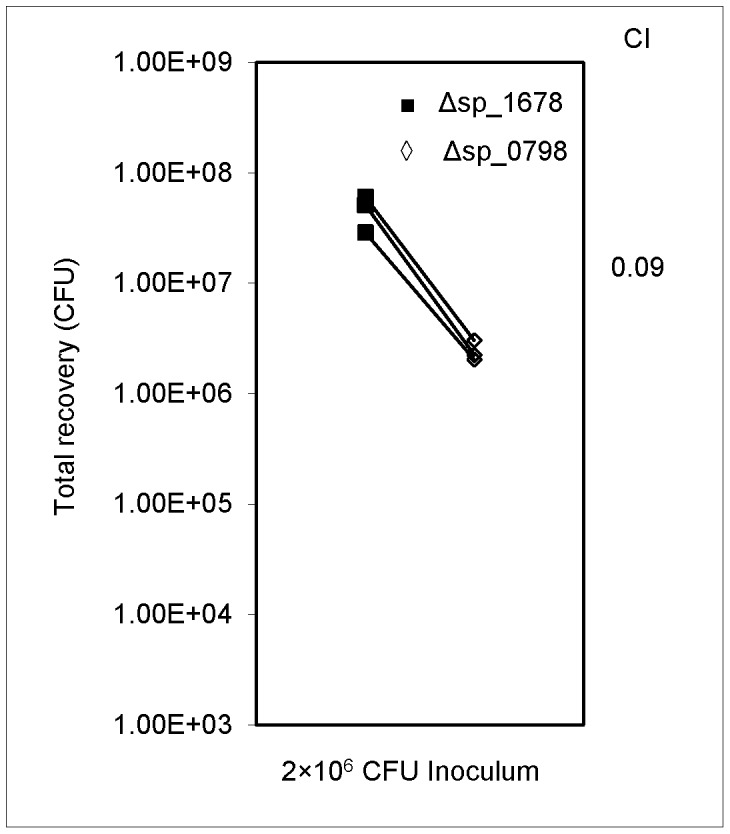
*In vivo* competitive index analyses of Δsp_0798. The CFU of both strains collected from vegetations are shown. The geometric mean CI value calculated for the Δsp_0798 mutant was 0.09. The statistical significance as determined by one-sample t test was *P*<0.01.

An additional competition experiment was performed to confirm the qPCR results. One candidate RR, SP_0798, also known as *ciaR,* was chosen for further testing based on the fact that the competitiveness of this mutant was significantly reduced in the rabbit endocarditis model. For comparison, a different antibiotic selection marker, a Km resistance cassette, was inserted to replace the SP_1678 gene. The control Δsp_1678_Km mutant and the Δsp_0798 mutant were co-inoculated in equal numbers in the IE animal model. The results from the analysis of three animals indicated that the Δsp_0798 mutant was significantly less virulent than the control strain (Fig. 2).

## Discussion

*S. pneumoniae* has been subject to much scrutiny due its prevalence as a cause of many serious diseases such as pneumonia, bacteremia, otitis media, meningitis, sinusitis, peritonitis and arthritis. However, its role in IE has been less studied. Genes involved in signal transduction through TCSs have been identified as virulence factors in models of other diseases such as pneumonia and meningitis in various studies [16]; however, none of these studies have included IE.

The primary goals of this study were to confirm that the *S. pneumoniae* TIGR4 strain was indeed capable of causing IE, to establish a quick screen using q-PCR for identification of virulence factors, and to identify virulence-related TCS of this strain. We established a system for constructing mutant strains by replacing target genes with an antibiotic-resistance cassette using PCR amplification and integrating this DNA into the genome by homologous recombination. We applied this method to obtain mutants lacking each of the TCS RR genes except the essential gene SP_1227. Behavior of all mutants was tested by mixing and inoculating them in the rabbit endocarditis model. Virulence was assessed by comparing bacterial abundance by quantitative real-time PCR and the CI assay. We found several mutants with decreased abundance in the rabbits and picked one candidate virulence factor in SP_0798 for further individual testing in the rabbit IE model. Not surprisingly given the different methods and control strains used in the two experiments, the CI and qPCR-CI values were not identical. However, both methods were in agreement in identifying the mutant as markedly and significantly reduced in virulence.

In previous studies of diseases such as pneumonia and bacteremia, many TCS in *S. pneumoniae* were reportedly involved in virulence. TCS01, TCS02, TCS04, TCS05, TCS06, TCS07, TCS08, TCS09, TCS12, TCS13, and TCS14 were found to have a role in virulence [16]. In our study of IE virulence, we found SP_0376/TCS14, SP_0798/TCS05, SP_1633/TCS01 and SP_2082/TCS04 were possible virulence factors based on statistical significance (P<0.01). Each of these RR proteins belongs to the Pho TCS family and has previously been identified as virulence factors of *S. pneumoniae* in other diseases, such as pneumonia and bacteremia [16].

We individually inoculated Δsp_0798 with our control mutant and confirmed that it did indeed have a role in virulence. SP_0798, known as CiaR, regulates many target genes. It has been shown to bind various promoter sites [25]. Microarray data also identified several genes that were significantly upregulated or downregulated by the mutant in comparison to the wild type. CiaR regulates genes with diverse functions including competence, antibiotic (beta lactam) resistance, stress responses, autolysis, polysaccharide metabolism and transport, and virulence [25]. Regulation of polysaccharide metabolism and transport is likely to have a role in endocarditis virulence as it would contribute to cell wall and capsule synthesis and composition. The cell wall and capsule protects the entire cell and therefore is important for the cell’s ability to survive any environment. Also, CiaR is found to regulate the *htrA* or high-temperature requirement A gene. It has previously been reported that in *S. pneumoniae* HtrA, also known as DegP/DO protease, confers tolerance to temperature shifts that pathogens often encounter. HtrA is a serine protease that can act as a molecular chaperone and has proteolytic activity. This protein usually serves as quality control with regard to other proteins in situations of high temperatures. In addition to heat resistance, HtrA can prevent protein degradation and assist in protein folding in situations of oxidative and osmotic stress [11]. The ability of HtrA to aid the cell in response to the environment through CiaR signaling may explain its role in virulence. Interestingly, five promoters strongly regulated by *ciaR* directed expression of small non-coding RNAs, otherwise known as *cia*-dependent small RNAs or csRNAs. It was reported that csRNA4 and csRNA5 affected stationary phase autolysis [26]. Another study reported that the CiaR target site affected biological peptide production including bacteriocin and cytolysin [25]. Bacteriocins are antimicrobial peptides that, when secreted, may inhibit or kill competing sensitive microbes or damage host defenses. Consequently, bacteriocin production may be advantageous for *S. pneumoniae* and contribute to growth and competitiveness in IE infection. Cytolysins are proteins secreted by bacteria that cause the death of other cells such as red blood cells through lysis. Therefore, the role of cytolysins in virulence may be similar to bacteriocins, in that they may also provide bacteria a growth advantage by inhibiting host defenses.

A study focused on Group B Streptococci (GBS) reported that *ciaR* mutants had significant decreases in intracellular survival in neutrophils, macrophages, and human brain microvascular endothelial cells. Additionally, these mutants were more susceptible to killing by antimicrobial peptides, lysozymes, and ROS generated by the immune system [27]. Although that study employed a different species than ours, it is possible that that *S. pneumoniae* may use similar mechanisms in hosts.

It is also possible that the main effect of the *ciaR* mutation is to reduce the *in vivo* growth rate. Given that the pathogenesis of streptococcal IE occurs primarily through enlargement of the vegetation due to bacterial growth [6], [28], a decrease in growth would be expected to have a profound effect on virulence. A complete and accurate understanding of the pathways regulated by such virulence factors as CiaR is of particular interest to public health as this information may lead to the identification of improved methods for combating infectious bacteria.

In conclusion, this study demonstrated that the *S. pneumoniae* TIGR4 strain caused IE in a rabbit model. A number of TCS RRs of *S. pneumoniae* TIGR4 strain contributed to virulence. Further experiments will be needed to elucidate how TCS are involved in IE as virulence factors, as well as the role of genetic regulation in virulence. Identification of regulatory networks of these TCSs may reveal novel signaling pathways related to IE and lead to better methods of prevention or treatment of the disease. Taken together, these results will bring more attention to *S. pneumoniae* as a causative agent for IE and may lead to more research on the subject.
